# Allergic diseases and Meniere's disease: a bidirectional Mendelian randomization

**DOI:** 10.1016/j.bjorl.2024.101472

**Published:** 2024-07-20

**Authors:** Hongru Qin, Meng Huang, Weiming Liang, Guojing Wu, Mengjia Tan, Junli Zhang, Wenyong Chen

**Affiliations:** Guangdong Provincial Hospital of Traditional Chinese Medicine, Department of Otolaryngology, Guangzhou, Guangdong, China

**Keywords:** Allergic diseases, Asthma, Allergic rhinitis, Eczema, Meniere's disease, Mendelian randomization

## Abstract

**Objectives:**

Allergic diseases and Meniere's disease found to have a possible link in observational study. However, the potential causal relationship between the two is unclear. Therefore, we aimed to explore the causal relationship between allergic diseases and Meniere's disease using a new data analysis technique called bidirectional Mendelian randomization study.

**Method:**

Summary-level statistics for Meniere's disease and three allergic diseases (asthma, allergic rhinitis, eczema/dermatitis) were obtained from large-scale genome-wide association studies. The inverse variance weighted method was used as the primary measure, supplemented by MR-Egger regression and the weighted median method. To ensure the reliability of the conclusions, Cochran's Q, MR-Egger intercept, MR-PRESSO test, leave-one-out test, and MR Steiger test were used.

**Results:**

Inverse-variance weighted method showed asthma (*p* = 0.008, OR = 3.908, 95% CI 1.424–10.724, adjust_*p* = 0.024), allergic rhinitis (*p* = 0.026, OR = 24.714, 95% CI 1.479–412.827, adjust_*p* = 0.026) and eczema/dermatitis (*p* = 0.019, OR = 3725.954, 95% CI 3.795 to 3,658,399.580, adjust_*p* = 0.029) all had a significant effect on Meniere's disease. Reverse Mendelian randomization studies have shown that Meniere's disease does not increase the risk of three allergic diseases. Sensitivity analysis showed no horizontal pleiotropy and heterogeneity for each trait.

**Conclusion:**

Our Mendelian randomization analysis supports a positive causal relationship between three allergic diseases (asthma, allergic rhinitis, eczema/dermatitis) and Meniere's disease. This suggests that physicians should pay more attention to the Meniere's patient's allergy history and consider allergy avoidance as part of their treatment plan.

**Level of evidence:**

Mendelian Randomized (MR) studies are second only to randomized controlled trials in terms of the level of evidence.

## Highlights

• From a genetic point of view.
• To provide new ideas for Meniere's treatment.
• A variety of MR analysis methods are used.
• A variety of sensitivity analysis methods are used.

## Introduction

Meniere's Disease (MD) is a chronic inner ear disease manifested by rotational vertigo, fluctuating sensorineural hearing loss, aural pressure, and tinnitus.^1^ According to the latest guidelines, the prevalence of MD fluctuates widely around the world, ranging from 3 to 513 cases per 100,000 people, with a peak age of onset in the 40 s.^2^ To date, there is no definitive theory to explain the pathophysiology of MD, and Endolymphatic Hydrops (ELH) is the pathological feature that best describes MD.^3^ MD is often described as a spontaneous multifactorial disorder with possible causes including structural dysfunction, immunologic damage, and genetic susceptibility.^4^ In recent years, more and more scholars have suggested that allergic reactions may be one of the risk factors for MD.^5^, ^6^, ^7^, ^8^ Although there are currently no Randomized Controlled Trials (RCTs) demonstrating that anti-allergic treatment can alleviate symptoms in patients with MD, an increasing number of cross-sectional studies suggest a possible association between allergic diseases and MD.

Allergies may be the potential cause in MD. This was first revealed by Duke^9^ in 1923 through case reports. Since then, attention has begun to focus on the association between allergic diseases and MD, and many observational experiments have demonstrated an epidemiologic overlap between the two diseases. An observational study showed that the prevalence of air allergies in MD patients was as high as 42%, and more than one-third tested positive for blood or skin allergies, which was significantly higher than in the control group with other ear diseases.^10^ In another prospective trial investigating the relationship between allergy therapies and MD, the symptoms of tinnitus and vertigo were significantly improved in MD patients treated with allergy immunotherapy and dietary elimination, implying that allergic responses may target the inner ear directly or indirectly.^5^ These investigations provide more evidence of a possible causal relationship between MD and allergy disorders. However, observational epidemiological research is vulnerable to reverse causality or confounding variables,^11^ and large-scale prospective RCTs are difficult to realise due to the low prevalence of MD. Therefore, we urgently need to find a new statistical method to explore the relationship between them.

MR refers to an analytical method used to identify the causality of an observed relationship between a modifiable exposures or risk factor and a clinically relevant outcomes.^12^ It is an application of the Instrumental Variables (IVs) approach, which uses genetic information as the IVs.^13^ Alleles for Single Nucleotide Polymorphisms (SNPs) are assigned to egg/sperm cells before any exposure or results, ensuring that results are less prone to reverse causal bias and confounding environmental exposures.^11^ Since the publication of large-scale Genome-Wide Association (GWAS) data, numerous studies have investigated the causal links between several variables using MR techniques. Two-Sample Mendelian Randomization (TSMR) is the process of analyzing data from two independent samples using MR methods. To date, there have been no studies using the TSMR approach to investigate the relationship between allergic diseases and MD.

Therefore, in order to investigate the role of allergies in the pathophysiology of MD and to offer guidance and a foundation for the clinical treatment of MD, we used the GWAS summary data to perform a two-sample bidirectional MR analysis to evaluate the causal relationship between three allergic diseases and MD.

## Methods

### Study design

**Fig. 1:**
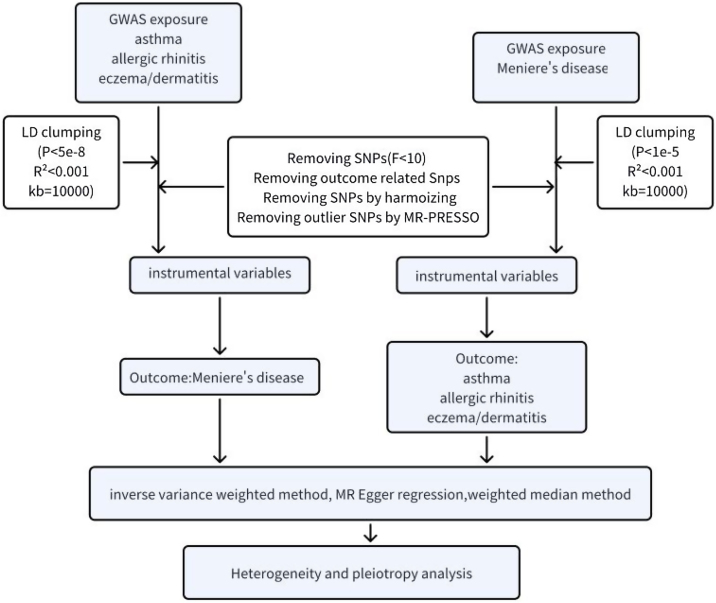
Flowchart of our bidirectional TSMR analysis. TSMR, Two-Sample Mendelian Randomization; GWAS, Genome-Wide Association; SNPs, Single, Nucleotide Polymorphisms; LD, Linkage Disequilibrium; MR-PRESSO, Mendelian Randomized; Pleiotropy Residual Sum and Outlier; MR-Egger, Mendelian Randomized Egger regression.

Our research aimed to investigate the relationship between allergic diseases and MD. In order to accomplish this, we performed a two-sample bidirectional MR analysis to examine the bidirectional causal effect between three allergic diseases (asthma, allergic rhinitis, eczema/dermatitis) and MD. This research method presumes the following three theoretical assumptions: (1) The IVs employed in the analysis should be significantly correlated with the exposure; (2) The IVs should be independent of confounding factors connected with the selected exposures and outcomes; (3) The IVs can only affect the results through the exposure and not through other pathways.^14^ The flowchart for this study is shown in Fig. 1.

### Data sources

The GWAS summary data for MD was obtained from the largest published GWAS study of MD in European populations,^15^ which included a total sample size of 482,774, including 1,526 cases and 481,248 controls. The MD data were obtained from the European Bioinformatics Institute (EMBI) and can be downloaded from the IEU open GWAS project (https://gwas.mrcieu.ac.uk/datasets/). Summarized GWAS data for asthma (53,257 cases and 408,756 controls), allergic rhinitis (26,107 cases and 436,826 controls) and eczema/dermatitis (11,819 cases and 451,114 controls) were collected from the UK Biobank (http://www.nealelab.is/uk-biobank). The asthma cases were identified from the doctor-diagnosed cases: those who answered “yes” to the question “Has a doctor ever told you that you have had any of the following conditions?” were classified as asthma cases. Data for allergic rhinitis and eczema/dermatitis were based on self-reports. The data selected for both exposure and outcome data are mixed-sex statistics of European ancestry. The data sources are detailed in Table 1.

**Table 1:** Details of GWAS included in the MR.

Trait	GWAS ID	Year	Nº of cases	Nº of controls	Number of SNPs
MD	ebi-a-GCST90018880	2021	1,526	481,248	24,194,682
Asthma	ukb-b-20296	2018	53,257	408,756	9,851,867
Allergic rhinitis	ukb-b-16499	2018	26,107	436,826	9,851,867
Eczema/Dermatitis	ukb-b-20141	2018	11,819	451,114	9,851,867

MR, Mendelian Randomization; MD, Meniere's Disease; GWAS, Genome-Wide Association; SNP, Single-Nucleotide Polymorphism.

### Instrumental variable selection

In the forward MR analysis, this study used P < 5e-8 as the standard to select SNPs related to exposure (asthma, allergic rhinitis and eczema/dermatitis). In the reverse MR analysis, SNPs associated with MD were selected based on P < 1e-5. The reason for selecting this threshold was to ensure the accuracy and testing power of TSMR analysis while including as many IVs as possible. To reduce bias induced by the Linkage Disequilibrium (LD) relationship between SNPs, this study selects independent SNPs using the “clump_data” function in the “TwoSampleMR” package in *R*. The screening criteria were: *R*^2^ = 0.001 and Clump Window = 10,000 KB. Weak IVs affect the results, so we use the F-test to assess whether a single SNP is affected by a weak instrumental variable. When the *F* statistic > 10, weak instrument bias can be overcome, and IVs can be ensured to be robust enough.^16^ F-statistic^17^ was calculated using the following formula: $\text{F}=R^{2}\times \left(\text{N}-2\right)/1-R^{2}$.

Here, N represents sample size and *R*^2^ is the variance of the risk of exposure explained by each instrument. *R*^2^ were calculated by the following equation^17^: ${\text{R}}^{2}=2\times \text{E}\text{A}\text{F}\times \left(1-\text{E}\text{A}\text{F}\right)\times {\text{b}\text{e}\text{t}\text{a}}^{2}/\left[2\right)\times \text{E}\text{A}\text{F}\times \left(1-\text{E}\text{A}\text{F}\right)\times {\text{b}\text{e}\text{t}\text{a}}^{2}+2\times EAF\times \left(1-EAF\right)\times N\times SE\left({\left(beta\right)}^{2}\right]$.

Here, beta is used to indicate the genetic estimate of exposure for each SNP. EAF stands for effect allele frequency, and SE for the standard error of the genetic effect.

In this study, we further examined if selected SNPs were linked to possible risk factor such as migraine using PhenoScanner, a comprehensive web-based database of genotypic phenotypic associations. SNPs associated with potential risk factors will be removed at the genome-wide significance level (*p* < 5 × 10‒8).

### MR analysis

Three MR analysis methods were used in this study: the inverse Variance Weighted (IVW) method, the Mendelian Randomized Egger regression (MR-Egger) method, and the Weighted Median (WM) method to assess the relationship between allergic diseases and MD. Among them, the IVW method was the primary analytical technique. The IVW method calculates a weighted average of the Wald ratios for individual SNPs based on the assumption that all tools are valid, so that the IVW method has maximum efficacy only when horizontal multidimensionality is balanced or absent.^18^ When all instrumental variables are invalid, the MR-Egger method is a potent tool since it is not dependent on the validity of the IVs.^19^ Even when up to 50% of the information contributing to the study comes from genetic variants that are invalid IVs, the WM method may produce a consistent estimate of the causal effect.^20^

### Sensitivity analysis

To validate the reliability of the MR analysis results, a variety of sensitivity analyses methods were used in this study, including Cochran’s Q test, MR-Egger intercept test, MR Pleiotropy Residual Sum and Outlier (MR-PRESSO), Leave-One-Out (LOO) analysis, MR Steiger test of directionality and Steiger filtering. The Cochran Q statistic is commonly used to assess heterogeneity, and if the *p*-value of the Cochran Q test is statistically significant (*p* < 0.05), the results are heterogeneous.^21^ In the MR-Egger test, the estimated intercept value can be taken as an estimate of the average pleiotropic effect across genetic variants. A large difference between the intercept value and 0, or a *p*-value of less than 0.05 for the MR-Egger test, suggests that these IVs may have horizontal pleiotropy.^19^ The MR-PRESSO method corrects for horizontal pleiotropy by detecting outliers and rejecting them.^22^ The LOO method is to use the remaining SNPs as instrumental variables for TSMR analysis after excluding a single SNP in turn to determine the effect of a single SNP on the total causal estimate. The MR Steiger directionality test is employed to evaluate the accuracy of the hypothetical causal direction. Steiger filtering is conducted to ascertain the correctness of the directionality of an individual SNP. The results of the interpretation of exposure by individual SNPs should be greater than outcome.^23^

### Quality control

The effect of multiple comparisons is controlled for using the error False Discovery Rate (FDR) correction, whereby the corrected value is designated as ‘adjust_P’. Should the ‘adjust_P’ of the IVW method be less than 0.05, this indicates that the probability of Type I Error occurring is less than 5%, thereby establishing a statistically significant result.^24^ All statistical analyses were performed in *R* (Version 4. 2.1). The results of this study are presented in the form of OR values and 95% CI, and the test level is α = 0.05.

## Results

### Genetic instruments

According to filter criteria, The IVs we chose are independently correlated with each exposure without linkage imbalance. The *F* value of IVs in allergic diseases is greater than 10 (Asthma: 1229.163, Allergic rhinitis: 1691.717, Eczema/dermatitis: 2305.717), which effectively eliminates the bias of weak instrumental variables on the results. In addition, the absence of SNPs has been found to be associated with a potential risk factor – migraine. In backward direction MR analysis, we removed the weak IVs (*F*<10) related with MD (rs144200851, rs146921001, rs146105719, rs117771503, rs7027172, rs117820341, rs112947850, rs192279461, rs75492200, rs141509041, rs139783790, rs73152856), which made the results less affected by weak instrument bias. Detailed instrumental variables information for each trait is provided in Supplement Tables S1‒4.

### MR analyses

In forward-direction MR analyses, we used allergic diseases as exposures to investigate the causal effect of allergic diseases on MD. Asthma was found to have a significant causal effect on MD (IVW, β = 1.363, *p* = 0.008, OR = 3.908, 95% CI 1.424–10.724, adjust_*p* = 0.024). Allergic rhinitis increased genetic susceptibility to MD (IVW, β = 3.207, *p* = 0.026, OR = 24.714, 95% CI 1.479–412.827, adjust_*p* = 0.026). The results of weighted median also supported this conclusion (WM, β = 4.412, *p* = 0.026, OR = 82.425, 95% CI 1.716–3959.608). MD was also linked to a genetic tendency to eczema/dermatitis (IVW, β = 8.223, *p* = 0.019, OR = 3725.954, 95% CI 3.795–3658399.580, adjust_*p* = 0.029).

**Fig. 2:**
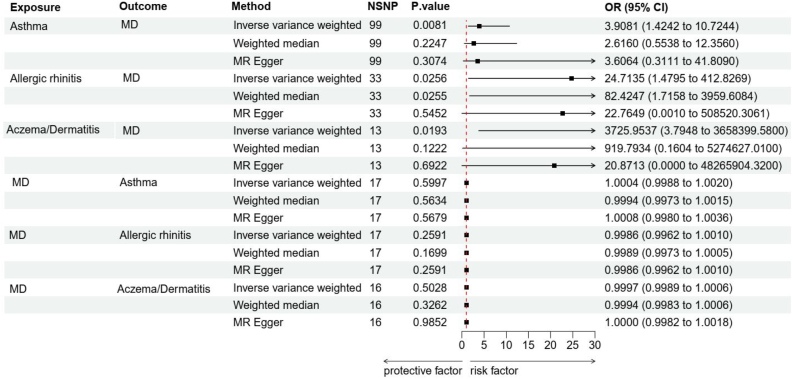
Forest plots of the causal relationship between three allergic diseases and MD. MD, Meniere's Disease; MR-Egger, Mendelian Randomised Egger regression; NSNP, Number of Single-Nucleotide Polymorphism; OR, Odds Ratio; CI, Confidence Interval.

In backward direction MR analyses, we used MD as an exposure to test the causal effect of MD on allergic diseases. However, there is no evidence that having MD increases the risk of the other three allergic diseases (asthma, allergic rhinitis, eczema/dermatitis) in all MR methods. Table 2 provides more details on the MR results. A forest plot of MR results is shown in Fig. 2. A summary scatter plot of the MR results is provided in Supplementary Fig. S1.

**Table 2:** MR analysis results of the bidirectional causal association between three allergic diseases and MD.

Exposure	Outcome	NSNPs	Method	βeta	p	OR	95% CI for OR		adjust_*p*
							Lower	Upper	
Asthma	MD	99	IVW	1.363	0.008	3.908	1.424	10.724	0.024
		99	WM	0.962	0.241	2.616	0.525	13.035	
		99	MR-Egger	1.283	0.307	3.606	0.311	41.809	
Allergic rhinitis	MD	33	IVW	3.207	0.026	24.714	1.479	412.827	0.026
		33	WM	4.412	0.026	82.425	1.716	3959.608	
		33	MR-Egger	3.125	0.545	22.765	0.001	508520.306	
Eczema/Dermatitis	MD	13	IVW	8.223	0.019	3725.954	3.795	3658399.580	0.029
		13	WM	6.824	0.122	919.793	0.160	5274627.010	
		13	MR-Egger	3.038	0.692	20.871	<0.001	48265904.315	
MD	Asthma	17	IVW	0.0004	0.600	1.000	0.999	1.002	/
		17	WM	−0.001	0.563	0.999	0.997	1.001	
		17	MR-Egger	0.001	0.568	1.001	0.998	1.004	
MD	Allergic rhinitis	17	IVW	−0.001	0.259	0.999	0.996	1.001	/
		17	WM	−0.001	0.170	0.999	0.997	1.000	
		17	MR-Egger	−0.001	0.259	0.999	0.996	1.001	
MD	Eczema/Dermatitis	16	IVW	−0.0002	0.503	1.000	0.999	1.001	/
		16	WM	−0.001	0.326	0.999	0.998	1.001	
		16	MR-Egger	0.00002	0.985	1.000	0.998	1.002	

MR, Mendelian Randomization; MD, Meniere's Disease; IVW, Inverse Variance Weighted; OR, Odds Ratio; MR-Egger, Mendelian Randomised Egger regression; WM, Weighted Median; CI, Confidence Interval; NSNP, Number of Single-Nucleotide Polymorphism.

### Sensitivity analyses

The MR-Egger model shows that there is no horizontal pleiotropy for each trait, (0.159 < intercept pval < 0.987) (Table 3). In the MR Steiger directionality test, the variance of the SNP explanations included in exposures was greater than the outcome, suggesting that the observed causal direction may be correct. In the Steiger filtering, 0 SNPs in all traits were found to be more predictive of outcome than itself, the direction of all SNPs is true. In addition, the Cochran's Q test pval was greater than 0.05 for each trait studied, indicating that the effect of heterogeneity on the results did not need to be considered. The MR-PRESSO study revealed no significant outliers (global test *p*-value >0.05) among the SNPs included in all the traits. Table 3 shows the sensitivity analysis results. The LOO sensitivity analysis found that no one SNP had a substantial impact on the overall causal effect of each trait. A summary picture of the results of the LOO method is detailed in Supplementary Figures S2–4.

**Table 3:** Sensitivity analysis of the bidirectional causal association between three allergic diseases and MD.

Exposure	Outcome	Heterogeneity test (method: IVW)		Pleiotropy test (method:MR-Egger)		MR Steiger direction	MR-PRESSO Global Test	
		Q	Q_pval	Intercept	pval		RSSobs	pval
Asthma	MD	84.652	0.830	0.001	0.944	TRUE	87.703	0.862 (raw, 0 outlier)
Allergic rhinitis	MD	17.769	0.980	0.0004	0.987	TRUE	23.771	0.929 (raw, 0 outlier)
Eczema/Dermatitis	MD	15.977	0.192	0.020	0.447	TRUE	22.449	0.259 (raw, 0 outlier)
MD	Asthma	24.748	0.074	0.001	0.510	TRUE	28.007	0.079 (raw, 0 outlier)
MD	Allergic rhinitis	23.631	0.098	0.000	0.944	TRUE	27.031	0.106 (raw, 0 outlier)
MD	Eczema/Dermatitis	11.805	0.694	−0.001	0.159	TRUE	13.623	0.704 (raw, 0 outlier)

MD, Meniere's Disease; IVW, Inverse Variance Weighted; MR-Egger, Mendelian Randomised Egger regression; MR-PRESSO, MR Pleiotropy Residual Sum and Outlier.

## Discussion

Both MD and allergy are chronic conditions that have a significant impact on patients' quality of life, and although a growing number of observational studies provide many reliable results on the link between allergic states and MD, direct evidence of a causal relationship between the two diseases is still lacking. This is the first study to use MR analysis to investigate at the link between multiple allergic diseases and MD. The results of this study show that three allergic diseases were positively correlated with the occurrence of MD.

The strong association between allergy and MD has been repeatedly demonstrated in human and animal studies: The effect of antigen stimulation on inner ear function in MD patients has been studied in several experiments,^25^, ^26^ and they have used Electrocochleography (EChoG) to monitor the inner ear response to antigen stimulation in MD patients, and the results show an increase in the Summating Potential/Action Potential (SP/AP) ratio of the affected ear after the experiment. Lasisi et al.^27^ studied the hearing function of allergy patients and found that these patients had a higher prevalence of inner ear disease than the general population. In addition to this, Singh et al.^28^ compared auditory function in AR patients with healthy controls using Otoacoustic Emission (OAE) and Auditory Brainstem Response (ABR) tests, and found that AR patients may have abnormal OAE and ABR similar to cochlear injury. In animal experiments, Uno et al.^29^ observed an increase in the negative sum potential on EChoG and the threshold of ABR in guinea pigs following allergen challenge, whereas in guinea pigs treated with blockers, these manifestations were not observed. This suggests that the auditory changes caused by the sensitized guinea pig's inner ear may be caused by type I allergies.

However, the underlying mechanisms between allergic status and MD remain unclear. Derebery and Berliner^6^ contributed an excellent article to the study of the relationship between MD and allergies. They proposed three possible mechanisms around the central idea of Endolymphatic Sac (ES) inflammation. First, the ES peripheral fenestration vessels allow antigen penetration, which stimulates mast cell degranulation in pericystic connective tissue.^29^ The resulting release of inflammatory mediators may lead to the toxic accumulation of metabolites by reducing the filtration capacity of the sac. The second possibility is that circulating immune complexes accumulate in ES-fenestrated blood vessels, causing inflammation and capillary leakage around the endolymphatic sac. A third possible mechanism is a viral antigen-allergy interaction. Viral antigens infected in childhood stimulate the Waldeyer ring with subsequent T-cell migration to the ES,^30^ leading to chronic inflammation that causes minor damage to ES absorption.

In the study of the immunopathologic link of MD and allergy, current research involves a variety of pathways and cytokines. Some studies^31^, ^32^ suggest that total Immunoglobulin E (IgE) may play a role in the pathogenesis of MD. In a study comparing allergen and IgE levels in patients with MD and healthy individuals, Roomiani et al.^32^ found that total serum IgE levels were significantly higher in patients with MD than in controls. Additionally, MD patients exhibited greater reactivity to inhalant and food allergens. Zhang et al.^31^ found that serum IgE levels were positively correlated with endolymphtic grade, hearing stage, and functional level, and found that CD23 expressed on hair cells can act as an IgE-binding receptor, so blocking CD23-mediated IgE transport may be a potentially important target for the treatment of MD. In addition, they detected IgE deposition in the ampulla, macula, semicircular canals, and ES of MD patients with high and low basal IgE levels, suggesting that IgE may not be produced by cyclic IgE translocation but by orthotopic production. By upregulating levels of IL-4, CD23 expression in HEI-OC1 cells and Vestibular End Organs (VEOs) can be increased, suggesting that elevated serum IL-4 may be responsible for increased CD23 expression and IgE deposition in MD. Keles et al.^8^ compared blood samples from MD patients and controls and found significant positive correlations between CD23 and IgE, CD8 and IgE, CD4/CD8 and IgE, and CD23 and CD8 in patient serum samples.

In addition to allergies, hypoxia and may also be associated with MD and serve as mediators of allergic disease associated with MD. Previous studies have shown that hypoxia is a potential physiological and pathological mechanism of asthma and allergic rhinitis.^33^, ^34^, ^35^, ^36^ One observational study suggests that hypoxic damage may lead to asymmetry in the peripheral vestibular system.^37^ Furthermore, a prospective research has demonstrated the efficacy of Continuous Positive Airway Pressure (CPAP) treatment in mitigating the symptoms of vertigo and low-frequency hearing loss associated with MD.^38^ Consequently, it makes sense to believe that hypoxia could be a risk factor for MD.

Notably, an observational study revealed that the prevalence of allergy history in patients with MD and migraine was significantly higher than in MD alone, suggesting that migraine may be a confounding factor between allergy and MD.^39^ Munno et al. tested cytokines in migraine patients and found that IL-5 and IL-4 levels were significantly elevated, which supports the immunoallergic mechanism of migraine. Therefore, when we remove confounders, we must ensure that the instrumental variables selected are not related to migraine, which makes the results of this study more credible.

There are some merits worth mentioning in this article: First, this study is the first to use the MR method to examine the connection between asthma, allergic rhinitis, eczema, and MD, saving time and economic costs while expanding the scope of traditional observational research. Secondly, the effect of migraine, a confounding factor, was excluded in order to minimize the effect of residual confounding and reverse causation. Third, a variety of MR sensitivity analysis methods were used in this study, and the consistency between sensitivity methods further supported the validity of the effect estimates.

However, our study has some limitations as well. First, dermatitis and eczema are complex skin conditions that may be affected by atopy, irritant, or allergic factors,^40^ but the dermatitis/eczema data involved in this study are not specifically stratified, so the results may also be influenced by others factors. Second, the samples are all from Europe, and the conclusions may be difficult to generalize to other ethnic groups. In addition, this study analyzed the relationship between only three allergic diseases and MD. There may also be a causal relationship between other allergic diseases and MD.

## Conclusion

In conclusion, our study demonstrates a causal relationship between allergic diseases and MD. Therefore, in the treatment of patients with MD, doctors should pay more attention to the patient's allergy history and consider avoiding allergy treatment as part of the treatment plan to help the patient manage MD symptoms. At the same time, the exact pathological mechanism behind causality needs to be explored through further experiments.

## Funding

This study was funded by the Key Discipline Construction Project of Chinese Medicine Talent Training from the Department of Otolaryngology, Guangdong Provincial Hospital of Traditional Chinese Medicine, Project No. 0102023701.

## Conflicts of interest

The authors declare no conflicts of interest.
