# Supplementary material for: Allergic diseases and Meniere's disease: a bidirectional Mendelian randomization
Source: Braz J Otorhinolaryngol. 2024 Jul 20;90(6):101472. doi: 10.1016/j.bjorl.2024.101472 (PMC11338938; doi:10.1016/j.bjorl.2024.101472)

**BJORL-D-24-00088 – Supplementary Material**

**Supplement Table 1** Instrument variables of asthma.

| **SNP** | **effect_allele** | **other_allele** | **βeta** | **se** | **pval** | ***F*** |
| --- | --- | --- | --- | --- | --- | --- |
| rs10068466 | T | C | -0.00503672 | 0.0007189 | 2.40E-12 | 1391.008 |
| rs10158467 | G | A | 0.00479973 | 0.000735742 | 6.90E-11 | 1359.166 |
| rs10486391 | G | A | -0.00464953 | 0.000674735 | 5.50E-12 | 1482.057 |
| rs11071559 | T | C | -0.00933366 | 0.000980948 | 1.80E-21 | 1019.418 |
| rs11088309 | G | C | 0.00690465 | 0.000944884 | 2.70E-13 | 1058.326 |
| rs11178649 | T | G | -0.00428923 | 0.000674123 | 2.00E-10 | 1483.402 |
| rs11185551 | C | G | -0.00502402 | 0.000860688 | 5.30E-09 | 1161.856 |
| rs117158080 | C | T | -0.00658859 | 0.00105872 | 4.90E-10 | 944.5327 |
| rs117552144 | T | C | 0.00936618 | 0.00141224 | 3.30E-11 | 708.0919 |
| rs117710327 | A | C | -0.0141041 | 0.00135267 | 1.90E-25 | 739.2754 |
| rs12123821 | T | C | 0.0172378 | 0.00155911 | 2.00E-28 | 641.3888 |
| rs12324931 | C | A | 0.00895133 | 0.00158339 | 1.60E-08 | 631.5536 |
| rs12788104 | G | A | 0.00512 | 0.00071535 | 8.20E-13 | 1397.911 |
| rs12939832 | A | G | -0.0108799 | 0.000662695 | 1.40E-60 | 1508.983 |
| rs12964116 | G | A | 0.0109934 | 0.00176 | 4.20E-10 | 568.1794 |
| rs1420466 | G | T | 0.00401102 | 0.000713901 | 1.90E-08 | 1400.748 |
| rs1552458 | G | A | -0.00444183 | 0.000720247 | 7.00E-10 | 1388.407 |
| rs1608554 | G | A | 0.00423569 | 0.000692123 | 9.40E-10 | 1444.824 |
| rs1655558 | G | T | 0.00422052 | 0.000669568 | 2.90E-10 | 1493.494 |
| rs1689510 | C | G | 0.00666899 | 0.000699137 | 1.40E-21 | 1430.329 |
| rs16903574 | G | C | 0.00995575 | 0.00127563 | 6.00E-15 | 783.923 |
| rs1696361 | C | T | -0.00417165 | 0.000691046 | 1.60E-09 | 1447.075 |
| rs17057868 | C | T | -0.00439029 | 0.000781321 | 1.90E-08 | 1279.878 |
| rs174535 | C | T | -0.00533434 | 0.000693582 | 1.50E-14 | 1441.784 |
| rs17454584 | G | A | 0.00709325 | 0.000801921 | 9.10E-19 | 1247 |
| rs1800440 | C | T | 0.00465738 | 0.000848743 | 4.10E-08 | 1178.208 |
| rs1837253 | C | T | 0.0111465 | 0.000753503 | 1.60E-49 | 1327.129 |
| rs191318 | A | G | -0.00421771 | 0.000713981 | 3.50E-09 | 1400.591 |
| rs2132404 | A | C | 0.00495462 | 0.000907004 | 4.70E-08 | 1102.526 |
| rs2197415 | G | T | 0.00986951 | 0.000670138 | 4.30E-49 | 1492.223 |
| rs2296618 | G | A | -0.00659692 | 0.000970135 | 1.00E-11 | 1030.78 |
| rs2299012 | C | A | 0.0101341 | 0.000836924 | 9.50E-34 | 1194.846 |
| rs2460555 | C | G | -0.00404086 | 0.000735572 | 3.90E-08 | 1359.48 |
| rs2477923 | C | T | -0.00477137 | 0.000664856 | 7.10E-13 | 1504.079 |
| rs2523668 | A | C | 0.00868103 | 0.000903266 | 7.20E-22 | 1107.089 |
| rs256866 | C | T | 0.00846627 | 0.00102535 | 1.50E-16 | 975.2725 |
| rs2671654 | G | A | -0.00551005 | 0.000671645 | 2.30E-16 | 1488.875 |
| rs274947 | C | T | -0.00397091 | 0.000662803 | 2.10E-09 | 1508.737 |
| rs2811387 | T | C | 0.0064071 | 0.00102831 | 4.60E-10 | 972.4652 |
| rs28498223 | T | C | 0.00589471 | 0.000739603 | 1.60E-15 | 1352.071 |
| rs2949669 | T | C | -0.00604271 | 0.000673249 | 2.80E-19 | 1485.328 |
| rs301816 | A | G | 0.00469359 | 0.000673829 | 3.30E-12 | 1484.05 |
| rs3024664 | C | T | 0.0125495 | 0.00140039 | 3.20E-19 | 714.0837 |
| rs3122929 | T | C | 0.00826019 | 0.000677054 | 3.10E-34 | 1476.981 |
| rs3181360 | T | C | -0.00870706 | 0.001594 | 4.70E-08 | 627.3499 |
| rs34173062 | A | G | 0.00799357 | 0.00135041 | 3.20E-09 | 740.5126 |
| rs34290285 | A | G | -0.0108097 | 0.000756848 | 2.80E-46 | 1321.264 |
| rs34712979 | A | G | 0.00430214 | 0.000758237 | 1.40E-08 | 1318.843 |
| rs35320232 | T | C | -0.00672714 | 0.00102483 | 5.20E-11 | 975.7674 |
| rs35570272 | T | G | 0.0058917 | 0.000679163 | 4.10E-18 | 1472.394 |
| rs35621564 | G | A | -0.00530401 | 0.000691599 | 1.70E-14 | 1445.918 |
| rs36045143 | G | A | -0.0089307 | 0.00076501 | 1.70E-31 | 1307.167 |
| rs3771180 | T | G | -0.0172533 | 0.000954039 | 4.20E-73 | 1048.171 |
| rs3856439 | T | C | -0.0059387 | 0.000700498 | 2.30E-17 | 1427.55 |
| rs3912397 | A | G | 0.00558009 | 0.000928877 | 1.90E-09 | 1076.564 |
| rs41283642 | T | C | -0.0118628 | 0.00184396 | 1.20E-10 | 542.3088 |
| rs41285280 | T | C | 0.00577255 | 0.000775066 | 9.50E-14 | 1290.207 |
| rs4594881 | T | G | -0.00423312 | 0.000698544 | 1.40E-09 | 1431.543 |
| rs4722758 | G | C | 0.00670837 | 0.000823024 | 3.60E-16 | 1215.026 |
| rs4739738 | A | G | -0.00725488 | 0.000689434 | 6.80E-26 | 1450.459 |
| rs4749894 | G | A | -0.00489541 | 0.000771265 | 2.20E-10 | 1296.566 |
| rs479844 | G | A | 0.00470584 | 0.00066491 | 1.50E-12 | 1503.956 |
| rs4842921 | A | G | -0.00439212 | 0.000677671 | 9.10E-11 | 1475.636 |
| rs4910165 | G | C | -0.00444007 | 0.000713457 | 4.90E-10 | 1401.62 |
| rs516263 | C | A | 0.0048081 | 0.000724139 | 3.10E-11 | 1380.944 |
| rs5743618 | A | C | -0.00770688 | 0.000773982 | 2.30E-23 | 1292.014 |
| rs58029167 | G | A | 0.00581838 | 0.000745897 | 6.20E-15 | 1340.662 |
| rs58873874 | C | T | 0.00812842 | 0.00135491 | 2.00E-09 | 738.0532 |
| rs6002379 | A | G | -0.00495956 | 0.000822048 | 1.60E-09 | 1216.469 |
| rs6011033 | G | A | 0.0045306 | 0.000785418 | 8.00E-09 | 1273.202 |
| rs616488 | G | A | -0.00390872 | 0.000700169 | 2.40E-08 | 1428.22 |
| rs61816766 | C | T | 0.0171959 | 0.00192184 | 3.60E-19 | 520.3324 |
| rs61957178 | T | C | 0.00537182 | 0.000901871 | 2.60E-09 | 1108.801 |
| rs62275576 | C | A | 0.00833992 | 0.00152358 | 4.40E-08 | 656.346 |
| rs62408227 | G | A | -0.00765037 | 0.000690822 | 1.70E-28 | 1447.545 |
| rs6498021 | G | T | -0.00554526 | 0.000966055 | 9.50E-09 | 1035.133 |
| rs6990534 | G | A | -0.00417487 | 0.00072614 | 9.00E-09 | 1377.139 |
| rs7097384 | A | G | 0.00387688 | 0.000664249 | 5.30E-09 | 1505.453 |
| rs7125066 | G | A | -0.00379604 | 0.000683398 | 2.80E-08 | 1463.27 |
| rs72743461 | A | C | 0.0116018 | 0.00077923 | 3.90E-50 | 1283.313 |
| rs73012738 | G | T | 0.0046973 | 0.000718703 | 6.30E-11 | 1391.389 |
| rs73033536 | C | T | -0.00707873 | 0.00117217 | 1.60E-09 | 853.1149 |
| rs73078636 | A | G | -0.005807 | 0.000986833 | 4.00E-09 | 1013.338 |
| rs73196739 | T | C | -0.00637113 | 0.000885594 | 6.30E-13 | 1129.181 |
| rs7555556 | T | C | -0.00483968 | 0.000702612 | 5.70E-12 | 1423.254 |
| rs76848919 | C | G | 0.00484317 | 0.000816501 | 3.00E-09 | 1224.733 |
| rs7734635 | G | A | 0.00856977 | 0.000910455 | 4.80E-21 | 1098.347 |
| rs7759971 | T | C | 0.0039325 | 0.000684797 | 9.30E-09 | 1460.28 |
| rs77634652 | C | G | 0.00501057 | 0.000907426 | 3.40E-08 | 1102.013 |
| rs7936312 | T | G | 0.0108852 | 0.000662015 | 9.50E-61 | 1510.533 |
| rs7992229 | A | G | 0.00638211 | 0.000722325 | 1.00E-18 | 1384.412 |
| rs8100197 | A | G | 0.00451375 | 0.000748482 | 1.60E-09 | 1336.032 |
| rs8103278 | A | G | -0.00424783 | 0.000694828 | 9.70E-10 | 1439.199 |
| rs9270911 | T | C | 0.0169719 | 0.000671823 | 8.30E-141 | 1488.481 |
| rs9277560 | G | C | -0.00646235 | 0.000922766 | 2.50E-12 | 1083.694 |
| rs9322952 | T | C | 0.00424185 | 0.000680066 | 4.40E-10 | 1470.439 |
| rs947591 | A | C | -0.00486072 | 0.000673715 | 5.40E-13 | 1484.301 |
| rs992969 | G | A | -0.013768 | 0.000762163 | 6.10E-73 | 1312.05 |
| rs9943 | G | A | -0.00424705 | 0.000694845 | 9.80E-10 | 1439.164 |

**Supplement Table 2** Instrument variables of allergic rhinitis.

| **SNP** | **effect_allele** | **other_allele** | **βeta** | **se** | **pval** | ***F*** |
| --- | --- | --- | --- | --- | --- | --- |
| rs1059513 | C | T | -0.00431566 | 0.000774669 | 2.50E-08 | 1290.868332 |
| rs11071557 | C | T | -0.00440732 | 0.00070754 | 4.70E-10 | 1413.341549 |
| rs11644510 | T | C | -0.00341817 | 0.000504636 | 1.30E-11 | 1981.617799 |
| rs118013485 | A | G | -0.00539037 | 0.000981301 | 3.90E-08 | 1019.050913 |
| rs12944882 | C | T | -0.00304863 | 0.000481574 | 2.40E-10 | 2076.515094 |
| rs1494564 | T | C | -0.00499528 | 0.000532805 | 6.90E-21 | 1876.851155 |
| rs17205373 | G | C | 0.00796641 | 0.000718614 | 1.50E-28 | 1391.561645 |
| rs17293632 | T | C | 0.00433923 | 0.00056427 | 1.50E-14 | 1772.193595 |
| rs1775554 | C | A | -0.00313874 | 0.000485227 | 9.90E-11 | 2060.882184 |
| rs1837253 | C | T | 0.00504538 | 0.000546306 | 2.60E-20 | 1830.468052 |
| rs2074585 | A | G | -0.00304187 | 0.000479118 | 2.20E-10 | 2087.159488 |
| rs2212434 | T | C | 0.00528633 | 0.000482564 | 6.30E-28 | 2072.255037 |
| rs2961920 | A | C | 0.00315081 | 0.000565392 | 2.50E-08 | 1768.676741 |
| rs2988277 | T | C | -0.00338427 | 0.000489802 | 4.90E-12 | 2041.632496 |
| rs3135020 | A | C | -0.00465183 | 0.00074864 | 5.20E-10 | 1335.749732 |
| rs34210653 | A | G | -0.0107444 | 0.00167316 | 1.30E-10 | 597.6688898 |
| rs34290285 | A | G | -0.00503463 | 0.000548757 | 4.50E-20 | 1822.292344 |
| rs346835 | T | C | -0.00386565 | 0.000509703 | 3.30E-14 | 1961.918372 |
| rs3856972 | G | A | 0.00287971 | 0.000494913 | 5.90E-09 | 2020.548419 |
| rs3892964 | C | A | -0.00316164 | 0.00056269 | 1.90E-08 | 1777.169809 |
| rs3939286 | C | T | -0.00511429 | 0.000551686 | 1.90E-20 | 1812.617467 |
| rs4233366 | T | C | 0.00307052 | 0.000541872 | 1.50E-08 | 1845.446304 |
| rs454875 | A | G | -0.00632059 | 0.00106617 | 3.10E-09 | 937.9326746 |
| rs5743618 | A | C | -0.00516897 | 0.000561006 | 3.10E-20 | 1782.504429 |
| rs59731494 | C | T | 0.00285276 | 0.000485117 | 4.10E-09 | 2061.349488 |
| rs6094570 | G | A | -0.00367513 | 0.000548298 | 2.00E-11 | 1823.81785 |
| rs6890286 | G | T | 0.0081106 | 0.000854579 | 2.30E-21 | 1170.161775 |
| rs7302200 | A | G | 0.00301261 | 0.000506448 | 2.70E-09 | 1974.527848 |
| rs73390208 | T | C | -0.0044582 | 0.000538583 | 1.30E-16 | 1856.716012 |
| rs763342 | C | A | 0.00275766 | 0.000505124 | 4.80E-08 | 1979.703359 |
| rs7688384 | T | C | -0.00428602 | 0.000513616 | 7.10E-17 | 1946.971433 |
| rs8067124 | T | A | 0.0114687 | 0.00183285 | 3.90E-10 | 545.5960279 |
| rs9807989 | C | T | -0.00513289 | 0.000528848 | 2.80E-22 | 1890.894321 |

**Supplement Table 3** Instrument variables of eczema/dermatitis.

| **SNP** | **Effect_allele** | **other_allele** | **βeta** | **se** | **pval** | ***F*** |
| --- | --- | --- | --- | --- | --- | --- |
| rs10791824 | G | A | 0.00233621 | 0.00033208 | 2.00E-12 | 3011.309563 |
| rs12123821 | T | C | 0.0118889 | 0.000773565 | 2.60E-53 | 1292.710606 |
| rs12434485 | C | T | -0.00278352 | 0.000459776 | 1.40E-09 | 2174.962764 |
| rs12801831 | T | C | 0.00260016 | 0.000476017 | 4.70E-08 | 2100.756233 |
| rs1568681 | T | C | -0.00254898 | 0.000394744 | 1.10E-10 | 2533.276452 |
| rs20541 | G | A | -0.00330046 | 0.000425177 | 8.30E-15 | 2351.951492 |
| rs2451258 | T | C | -0.00198127 | 0.000339671 | 5.40E-09 | 2944.012529 |
| rs28383392 | G | A | -0.00336971 | 0.000429986 | 4.60E-15 | 2325.647067 |
| rs6089970 | T | C | -0.00316548 | 0.000394681 | 1.10E-15 | 2533.68082 |
| rs61816766 | C | T | 0.0178279 | 0.000953391 | 5.00E-78 | 1048.883071 |
| rs62626322 | G | T | 0.00406764 | 0.000519295 | 4.80E-15 | 1925.679392 |
| rs7757906 | G | A | -0.00209524 | 0.00037257 | 1.90E-08 | 2684.047776 |
| rs9267947 | G | A | -0.00232789 | 0.000328147 | 1.30E-12 | 3047.40156 |

**Supplement Table 4** Instrument variables of Meniere's disease.

| **SNP** | **effect_allele** | **other_allele** | **βeta** | **se** | **pval** | ***F*** |
| --- | --- | --- | --- | --- | --- | --- |
| rs112518661 | T | C | 0.3111 | 0.0665 | 2.91E-06 | 15.03752889 |
| rs12240514 | A | G | 0.3826 | 0.084 | 5.26E-06 | 11.90471037 |
| rs13012831 | C | T | -0.1372 | 0.0308 | 8.58E-06 | 32.46739192 |
| rs17004339 | C | T | -0.2226 | 0.0493 | 6.38E-06 | 20.28388785 |
| rs17017756 | C | T | 0.1743 | 0.0371 | 2.63E-06 | 26.95406122 |
| rs17062478 | G | A | 0.211 | 0.0409 | 2.55E-07 | 24.44977191 |
| rs2148360 | C | T | 0.2537 | 0.0562 | 6.35E-06 | 17.79351728 |
| rs2150804 | C | T | -0.3199 | 0.0712 | 7.01E-06 | 14.04488302 |
| rs4783079 | A | C | -0.1342 | 0.03 | 7.71E-06 | 33.33318904 |
| rs60165883 | A | G | 0.2255 | 0.0477 | 2.25E-06 | 20.96426983 |
| rs602160 | C | A | 0.1828 | 0.0414 | 9.99E-06 | 24.15448481 |
| rs62004645 | T | C | 0.3598 | 0.0789 | 5.11E-06 | 12.67421636 |
| rs7038010 | T | C | 0.2092 | 0.043 | 1.15E-06 | 23.25571328 |
| rs7152656 | G | A | -0.1418 | 0.0316 | 7.31E-06 | 31.64543263 |
| rs72798118 | A | T | 0.4237 | 0.0942 | 6.90E-06 | 10.6156653 |
| rs74448956 | C | T | 0.285 | 0.0586 | 1.14E-06 | 17.06477254 |
| rs969201 | C | A | 0.1266 | 0.0285 | 9.04E-06 | 35.08756741 |

**Supplement Figure 1** Scatterplot of the causal relationship between three allergic diseases and Meniere's disease.


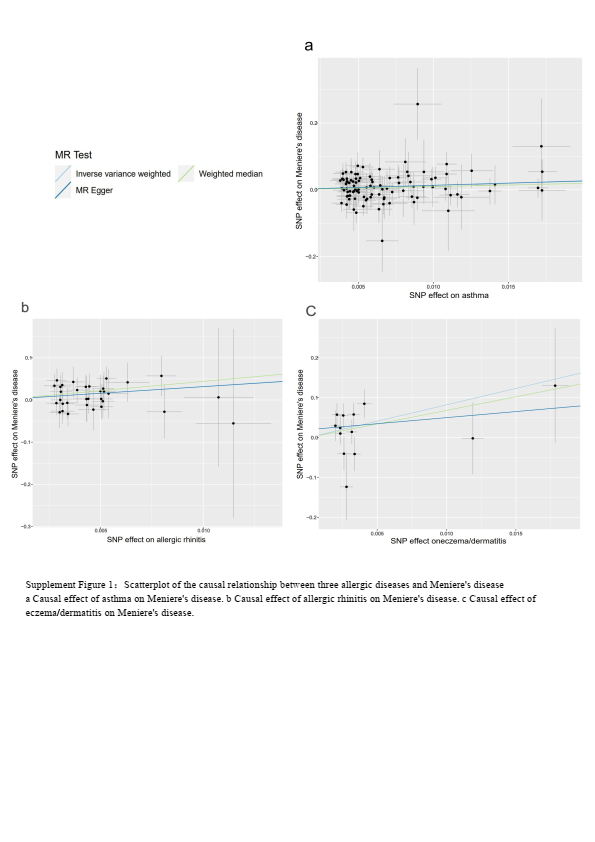


**Supplement Figure 2** Leave-one-out analysis for asthma on Meniere's disease.


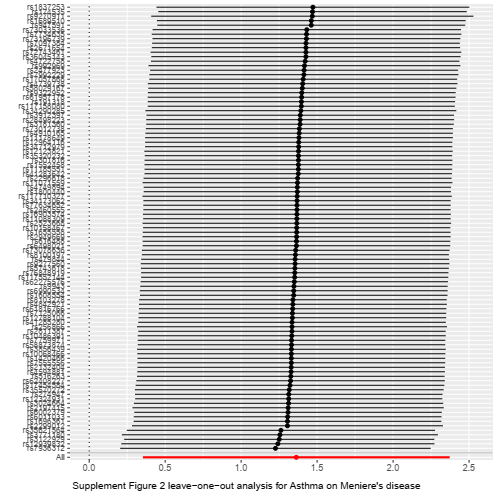


**Supplement Figure 3** Leave-one-out analysis for allergic rhinitis on Meniere's disease.


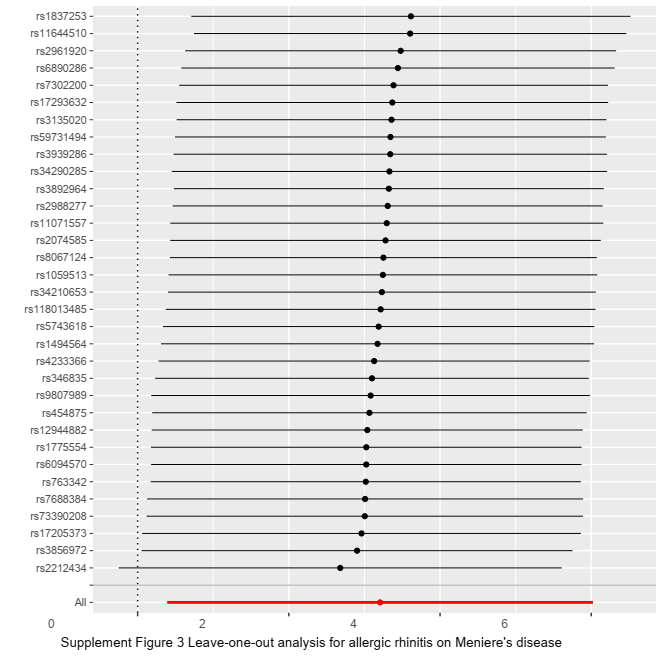


**Supplement Figure 4** Leave-one-out analysis for eczema/dermatitis on Meniere's disease.


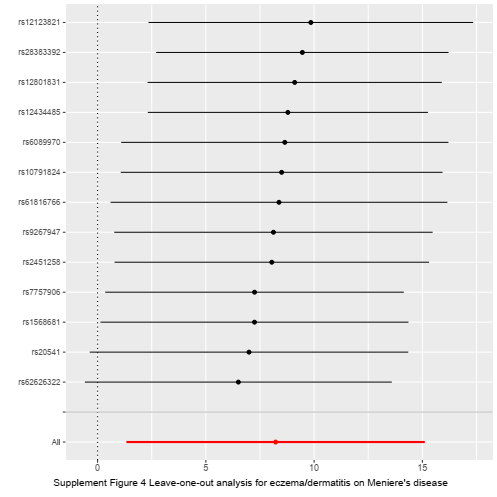

Supplement: Supplementary file 1 [file mmc1.docx]
